# Systematic review on Marburg virus prevalence and persistence in animals

**DOI:** 10.3389/fvets.2026.1756506

**Published:** 2026-02-04

**Authors:** Theophilus Odoom, Philip El-Duah, Rexford Mawunyo Dumevi, Therese Muzeniek, William Tasiame, Richard Kwamena Abbiw, George Agyei, Sherihane Aryeetey, John Amuasi, Faleke Olufemi Oladayo, Raphael Folitse, Benjamin Emikpe, Walter Bruchhausen, Christian Drosten

**Affiliations:** 1 German-West Africa Center for Global Health and Pandemic Prevention, Kumasi, Ghana; 2 School of Veterinary Medicine, Kwame Nkrumah University of Science and Technology, Kumasi, Ghana; 3 Accra Veterinary Laboratory, Accra, Ghana; 4 Institute of Virology, Charité - Universitätsmedizin Berlin, Corporate Member of Freie Universität Berlin, Humboldt-Universität Zu Berlin, and Berlin Institute of Health, Berlin, Germany; 5 West African Centre for Cell Biology of Infectious Pathogens, University of Ghana, Accra, Ghana; 6 Department of Clinical Microbiology, Kwame Nkrumah University of Science and Technology, Kumasi, Ghana; 7 Kumasi Centre for Collaborative Research in Tropical Medicine, Ghana, and Bernhard Nocht Institute for Tropical Medicine, Kumasi, Ghana; 8 Universitätsklinikum Bonn, Bonn, Germany

**Keywords:** bats, livestock, Marburg virus disease, MARV antibodies, MARV

## Abstract

**Introduction:**

Marburg Virus Disease (MVD) is a fatal zoonotic disease of humans and nonhuman primates caused by the Marburg virus (MARV) of the *Filoviridae* family, and presenting as hemorrhagic fever with a high fatality rate. Egyptian fruit bats, *Rousettus aegyptiacus*, are the principal natural reservoir, with evidence linking them to most human outbreaks.

**Methods:**

This systematic review evaluated the prevalence of MARV in bats, domestic animals, and rodents, as well as the duration of antibodies and potential routes of viral shedding. A comprehensive search of six (6) scientific databases identified 30 studies meeting the inclusion criteria.

**Results:**

In bats, seroprevalence ranged from less than 1% to about 54% while MARV genes were detected in 0.8–3% of samples. MARV antibodies persisted for up to 11months in naturally infected bats, while induced or maternal antibodies declined within 5 months. Apart from *Rousettus aegyptiacus*, occasional seropositivity was detected in other bat species such as *Epomops franqueti*, *Micropteropus pusillus*, *Hypsignathus monstrosus*, and *Eidolon helvum*, whereas MARV particles were observed in *Rousettus aegyptiacus* and *Hipposideros* spp. Even though viral genes were undetected in domestic animals, non-human primates (NPH) and rodents, antibodies were reported in dogs and livestock, and NPH in Ghana and Gabon and Zambia, respectively, indicating a higher probability of non-lethal MARV exposure in these species.

**Discussion:**

These findings confirm *Rousettus aegyptiacus* as the primary reservoir but suggest that other bats and domestic animals may contribute to the natural maintenance of MARV. Expanded multispecies surveillance in high-risk regions is essential to clarify reservoirs, host distribution, and transmission dynamics. Understanding these patterns is critical for designing targeted interventions to reduce spillover risk to humans.

## 1 Introduction

Marburg Virus Disease (MVD) is a highly pathogenic zoonotic disease affecting both humans and non-human primates (1). It is caused by the Marburg virus (MARV) of the *Filoviridae* family under the genus *Orthomarburgvirus* (2). The virus is classified as a Category A and Risk Group 4 pathogen by the World Health Organization (WHO) and the Centers for Disease Control and Prevention (CDC) (3), and it is considered to have pandemic potential, posing a significant threat to global health security (4). MVD manifests as a hemorrhagic fever syndrome and multiple organ failure in humans (5, 6). The disease has an average fatality rate of approximately 50% (ranging from 24 to 88%), depending on the outbreak’s severity and the quality of healthcare provided during the outbreak (7). In the absence of a known cure, patient management is restricted to symptomatic and supportive care, along with the treatment of concomitant complications (8).

The Egyptian fruit bat, *Rousettus aegyptiacus*, is identified as the natural reservoir of the MARV, as evidenced by serological and molecular findings in bats (9, 10), and its association with at least 80% of all human MVD outbreaks (11). Although the epidemiology of MVD is not fully understood, the distribution of MARV appears to align with the geographical range of its reservoir bats (6, 11, 12). Furthermore, MVD outbreaks in human populations are significantly influenced by anthropogenic activities, such as contact with infected animals in laboratory settings or after extended periods in caves (7). With the exception of the United States, Germany, the Netherlands, and Russia (Koltsovo, Soviet Union), all reported MVD outbreaks have occurred in Africa (13), suggesting that the disease is predominantly an African issue. Most of these outbreaks lack epidemiological links and have been sustained either through continuous human-to-human transmission following an initial spillover event, as observed in Angola between 2004 and 2005 (3, 14), or through repeated introductions of the virus, as seen in the Democratic Republic of Congo (5). In this review, we examined the prevalence of MARV in bats, rodents, non-human primates (NHP) and domestic animals from the first recorded outbreak to May 2025. We also reviewed the duration of antibodies, and the routes of viral shedding by the selected identified species.

## 2 Methods

We followed the guidelines of the Preferred Reporting Items for Systematic Reviews and Meta-Analyses (PRISMA) 2020 expanded checklist (Supplementary file 1) (15). The review questions were formulated using the CoCoPop framework (Condition, Context, Population) (16). The conditions of interest were MARV infection, the duration of MARV antibody persistence in exposed animals, and potential transmission routes. The population comprised bats, rodents, NHP, and domestic animals, and the context encompassed both wild and domestic settings. Population, Intervention, Comparator, and Outcome (PICO)–based framework was not applied, as this has been associated with reduced recall in non-interventional reviews (17). As this review did not evaluate an intervention or comparator, application of the PICO framework was considered methodologically inappropriate. Accordingly, the CoCoPop framework was adopted as the most appropriate and rigorous structure to guide the review questions, eligibility criteria, and search strategy. This review was not registered in PROSPERO because, at the time of protocol development, the review type “incidence or prevalence of a disease or condition” was not available for registration for systematic reviews involving animals as the primary study population.

### 2.1 Search strategy and selection

To identify pertinent articles, we customized our search strategy to align with the requirements of six scientific databases and search engines, namely PubMed, Google Scholar, Web of Science, African Journal Online (AJOL), Scopus, ScienceDirect and other gray literature. The search employed the following keywords: “Marburg virus,” “Marburg virus disease,” “bat,” “livestock,” “domestic animals,” “non-human primate,” “dog,” “cattle,” “sheep,” “goat,” “filovirus,” “Rousettus” and “prevalence.” Additionally, Medical Subject Headings (MeSH) terms related to Marburg disease, specifically “Marburgvirus,” “Marburg Disease,” “Filoviridae,” and “Hemorrhagic Fever, Viral,” were utilized to retrieve relevant publications from PubMed. The search was restricted to English-language articles published before May 2025, and excluded preprints, reviews, and meta-analyses. The initial search was conducted on March 18, 2025, and subsequently repeated on April 26, 2025. All retrieved publications were uploaded to Mendeley Reference Manager Version 2.130.2. Duplicates were identified using Mendeley and manually verified by comparing titles, authorship, and DOI.

The remaining articles were screened by title and abstract based on predetermined inclusion criteria. A publication was selected if it was written in English and investigated the prevalence of MARV in bats, rodents, NHP or livestock. During the screening process, publications not meeting the initial criteria were excluded. Prevalence was categorized as molecular (MP) if PCR- or sequence-based methods were employed, and serological (SP) if ELISA, Immunofluorescence, Bio-Plex, Luminex or other serology assays were utilized. For screening purposes, the term livestock encompassed all domesticated mammals, including pigs, goats, sheep, and cattle. Although the primary focus was on the aforementioned criteria, studies on the duration of MARV antibodies and the isolation and shedding of MARV in bats and livestock were also included. Full texts of all accepted publications were obtained and independently assessed for inclusion by two independent reviewers (ARK and TO). All disagreements were resolved by a third reviewer (PED).

### 2.2 Data extraction and analysis

KoboToolbox was used to develop a questionnaire that formed the basis for extracting information from the accepted publications (Supplementary file 2). The questionnaire collected metadata on the publications, species being investigated, diagnostic approach(es) used, and Joanna Briggs Institute (JBI) risk of bias assessment (ROB). Based on the JBI ROB assessment, studies were included, excluded, or referred to a third author for additional evaluation. Other useful information that provided valuable context to extracted data including effect of sex and age on MARV prevalence were collected. Unclear information was clarified by other authors and only accepted if a consensus was reached. The data were exported to CSV file and analyzed using RStudio Version 2024.12.0 Build 467. A mixed-effect meta-regression model was fitted using restricted minimum likelihood estimation, with study year, serological testing, and country included as moderators. Heterogeneity was quantified using τ^2^, I^2^, and H^2^, and the proportion of heterogeneity explained by moderators was assessed with R^2^. Residual heterogeneity was tested with Cochran’s Q statistic. Descriptive statistics were presented as plots and tables, and all inferential statistics were presented as CSV file (Supplementary file 2).

### 2.3 Ethical consideration

This study involved the analysis of already published work and did not involve primary data or human or animal participants. Therefore, ethical approval was not required. However, the authors took intentional steps to prevent the misrepresentation of information obtained from the publications used in this systematic review. These steps included strict inclusion and exclusion criteria, transparent reporting using the PRISMA checklist, use of reference management software and avoiding selective reporting.

## 3 Results

**Figure 1:**
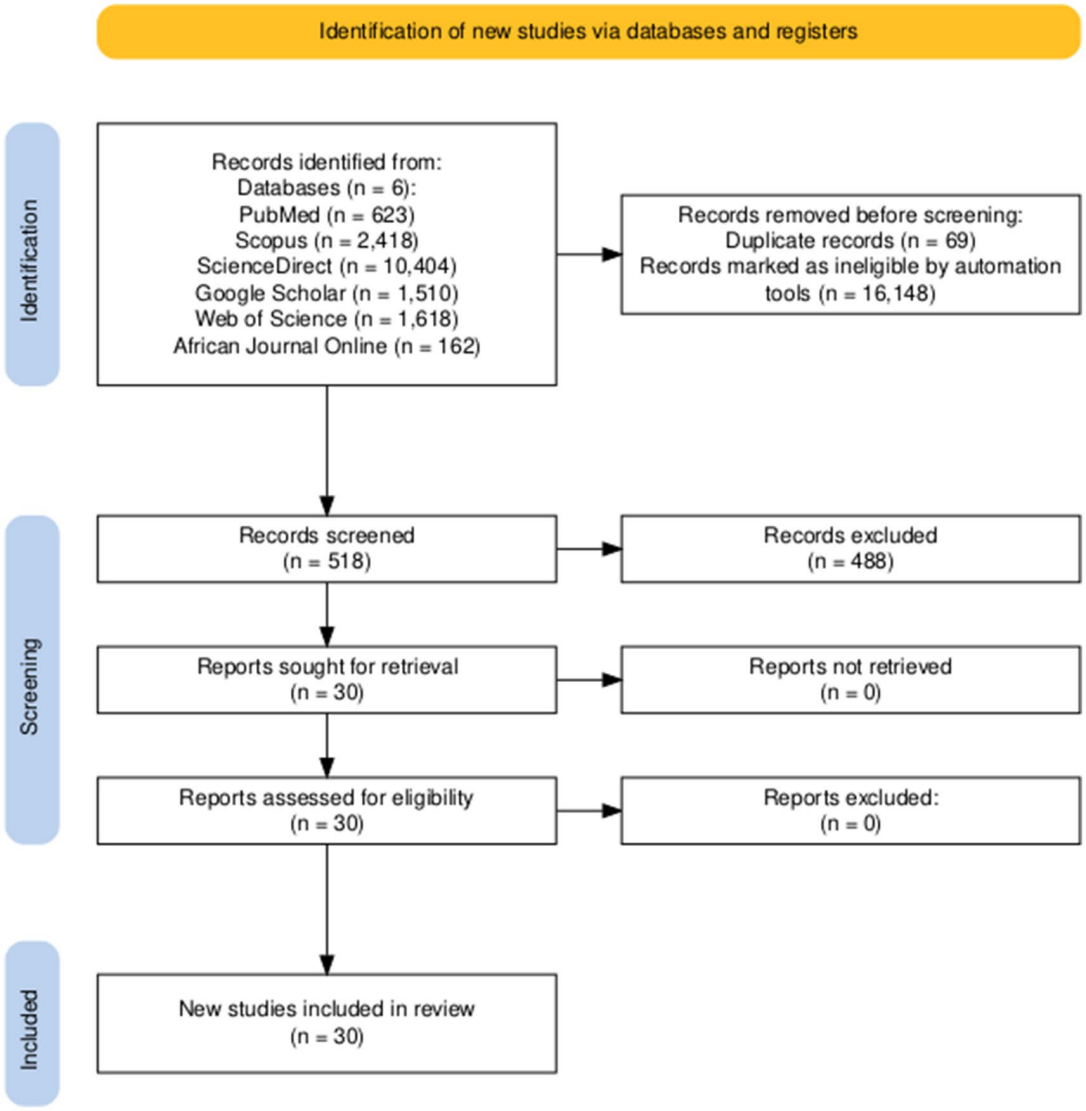
Identification of relevant studies via databases and registers.

A total of 16,735 publications were obtained from the initial search. After applying the predefined filters (publication year and article type) and removing 69 duplicate records, 518 publications were screened; of which 488 were excluded based on the eligibility criteria (Figure 1). Papers were excluded based on three major reasons; reports of published studies by other sources, e.g., CDC (0.2%; 1/488), preliminary reports of studies prior to the publication of the study (0.2%; 1/488), and studies that did not meet the inclusion criteria (99.6%; 486/488). The full texts of the remaining 30 articles were retrieved and evaluated. None of the retrieved articles were excluded after evaluation (Figure 1). Prevalence of MARV was discussed in 26 articles; thus, bats (22), domestic animals (3), NHP (1) and rodents (2). Five publications provided insights into the duration of MARV antibodies and isolation and shedding of MARV in bats.

Only 30 records met the inclusion criteria after successive screening processes. A record was included in the study if it was in English, a full article, investigated the prevalence of MARV RNA and antibodies, provided insights into the duration of MARV antibodies, or isolation or shedding routes of MARV RNA in bats and other animals. The PRISMA diagram was generated using the PRISMA Flow Diagram tool (18).

### 3.1 Prevalence of MARV in bats

Of the 22 articles on the prevalence of MARV-specific antibodies in bats, 19/22 (86.36%) investigated the SP of MARV antibodies in bats, while 21/22 (95.45%) used various PCR-based methods to amplify the Viral Protein 35 (VP35), VP30, VP24, Nucleoprotein (NP), Large polymerase (L) or other pan-filoviral genes of MARV. While blood is the preferred sample for serology, liver, spleen, and oral and fecal swabs are the preferred samples for MARV RNA demonstration (Supplementary file 2).

The articles described MARV surveillance in bat populations across cave systems and roosting sites in 14 countries: Democratic Republic of Congo, Gabon, Democratic Republic of Congo (DR Congo), Guinea, India, Kenya, Nigeria, Sierra Leone, South Africa, Singapore, Uganda, United States, Viet Nam, and Zambia. Only South Africa, Uganda, DR Congo, Kenya, and Zimbabwe had officially recorded incidences of Marburg in humans. The SP recorded in bats ranged from 0.93 to 53.04%. Seropositive bat species identified were predominantly *Rousettus aegyptiacus* and rarely *Rhinolophus eloquens, Rousettus aegyptiacus, Epomops franqueti, Hypsignathus monstrosus, Micropteropus pusillus, Rousettus leschenaultii, Rousettus amplexicaudatus, Eonycteris spelaea, Cynopterus sphinx*, and *Eidolon helvum* (Table 1). Only two studies performed further studies to improve the confidence in the specificity of identified antibodies using paired longitudinal samples testing (19) and Western blot (20). The duration of MARV antibodies in about 84% of naturally infected *R. aegyptiacus* was estimated to be at least 11 months, 4 months in about 67% of experimentally infected bats, and maternal antibodies were lost in 84% of captured *R. aegyptiacus* juveniles within 5 months (21). It has also been established that bats can lose induced MARV-specific antibodies within 3 months (22).

**Table 1:** Prevalence of Marburg virus RNA and antibodies in bats.

Country	Serological testing in bats				PCR testing in bats				Species testing negative for MARV RNA and/or antibodies	Ref
	Total	Positive	SP (%)	Species positive	Total	Positive	MP (%)	Species positive		
Democratic Republic of Congo	426	52	12.21	*Rhinolophus eloquens*, *Rousettus aegyptiacus*	381	12	3.15	*Miniopterus inflatus, Rhinolophus eloquens*, *Rousettus aegyptiacus*	3 species of fruit bats (Megachiroptera) and 12 species of insectivorous bats (Microchiroptera), including *Chaerephon pumila, Rousettus aegyptiacus, Mops condylurus, Hipposideros caffer*, 11 other species.	(23)
Gabon	-	-	-	*-*	1,257	9	0.72	*Rousettus aegyptiacus*		(53)
Gabon, Republic of Congo	1,876	25	1.33	*Epomops franqueti, Hypsignathus monstrosus, Micropteropus pusillus, Rousettus aegyptiacus*	283	4	1.41	*Rousettus aegyptiacus*	*Myonycteris torquata, Microchiroptera, Megaloglossus woermanni, Eidolon helvum, Casinycteris argynnis*	(54)
Gabon, Republic of Congo	438	29	6.62	*Rousettus aegyptiacus*	1,138	4	0.35	*Rousettus aegyptiacus*	*Megaloglossus woermanni, Micropteropus pusillus, Hypsignathus monstrosus, Epomops Franqueti, Hipposideros gigas, Myonicterus torquata, Eidolon helvum, Microchiroptere, Rousettus aegyptiacus*	(27)
Guinea	-	-	-	*-*	501	3	0.60	*Rousettus aegyptiacus*		(29)
India	46	0	0.00	*-*	46	0	0.00	*-*	*Eonycteris spelaea, Rousettus leschenaultii*	(55)
Kenya	-	-	-	*-*	159	0	0.00	-	*Rousettus aegyptiacus*	(37)
Kenya	-	-	-	*-*	272	1	0.37	*Rousettus aegyptiacus*	*Chaerephon pumilus, Scotophilus dingali*	(56)
Nigeria	279	0	0.00	*-*	-	-	-	-	*Eidolon helvum*	(57)
Sierra Leone	140	12	8.57	*Rousettus aegyptiacus*	1755	11	0.63	*Rousettus aegyptiacus*	*Rousettus aegyptiacus*	(31)
South Africa	1,431	759	53.04	*Rousettus aegyptiacus*	159	3	1.89	*Rousettus aegyptiacus*	*Rousettus aegyptiacus*	(19)
South Africa	423	143	33.81	*Rousettus aegyptiacus*	416	12	2.88	*Rousettus aegyptiacus*	*Rousettus aegyptiacus*	(32)
Singapore	409	0	0.00	*-*	-	-	-	*-*	*Eonycteris spelaea, Cynopterus brachyotis, Pteropus lucasi*	(58)
Uganda	1,155	13	1.13	*Rousettus aegyptiacus*	1,220	32	2.62	*Hipposideros* spp., *Rousettus aegyptiacus*	*Hipposideros* spp.*, Rousettus aegyptiacus*	(24)
Uganda	400	66	16.50	*Rousettus aegyptiacus*	400	53	13.25	*Rousettus aegyptiacus*	*Rousettus aegyptiacus*	(30)
Uganda	1,622	250	15.41	*Rousettus aegyptiacus*	1,622	40	2.47	*Rousettus aegyptiacus*	*Rousettus aegyptiacus*	(14)
USA	-	-	-	-	512	0	0.00	-	*Eptesicus fuscus, Lasiurus borealis, Lasiurus cinereus, Myotis leibii, Myotis lucifugus, Myotis septentrionalis, Perimyotis subflavus*	(59)
Viet Nam	227	36	15.86	*Rousettus leschenaultii, Rousettus amplexicaudatus, Eonycteris spelaea,* *Cynopterus sphinx*	248	0	0.00	*-*	*Eonycteris spelaea, Cynopterus sphinx, Cynopterus horsfieldi, Macroglossus sobrinus, Rhinolophus microglobosus, Hipposideros pomona, Hipposideros cineraceus, Rousettus amplexicaudatus, Rhinolophus chaseni, Rhinolophus pusillus, Taphozous melanopogon, Myotis hasseltii, Taphozous longimanus, Scotophilus kuhlii,* *Rousettus leschenaultii*.	(60)
Viet Nam	-	-	-	*-*	1131*	0	0.00	-		(38)
Zambia	-	-	-	*-*	71	2	2.82	*Rousettus aegyptiacus*		(33)
Zambia	748	7	0.94	*Eidolon helvum*	748	0	0.00	-	*Eidolon helvum*	(20)
Zambia	290	127	43.79	*Rousettus aegyptiacus*	290	0	0.00	-	*Rousettus aegyptiacus*	(61)

SP, Seroprevalence; MP, Molecular prevalence; Ref, Reference; MARV, Marburg virus; PCR, Polymerase chain reaction; RNA, Ribonucleic acid. ‘*’ Shows bat-derived samples for PCR instead of individual bat samples. ‘-‘shows no data was obtained for such parameter.

MARV genes were amplified in <1–13.25% of sampled *Rousettus aegyptiacus* in DR Congo, Gabon, Guinea, Kenya, Sierra Leone, South Africa, Uganda, Zambia. In two instances, MARV RNA was demonstrated in *Miniopterus inflatus (1/33), Rhinolophus eloquens (7/197),* in DR Congo (23), and *Hipposideros* spp. (1/609) in Uganda (24) (Table 1). There is a lack of consensus on the viremic nature and duration in bats post-infection with MARV. For instance, while viremia was detected transiently (21, 25–27), the hypothesis of an existent long-term protective immunity for MARV infection in bats makes viremia undetectable (14, 21). This was used as a basis for the absence of viremia in most bats. The latter phenomenon was attributed to mechanisms other than antibody-mediated viral neutralization (28).

Study-level factors including country and ROB scores did not explain variation in MARV SP and MP, with substantial unexplained heterogeneity and potential small effects across studies (Supplementary file 2). Additionally, clear evidence of publication bias was not observed.

### 3.2 Circulating MARV isolates in bats

MARV has been isolated from several bat tissues including liver, spleen, lymph nodes, kidneys, lungs, stomach, feces, oral fluid and skin (14, 24, 29–31). All isolates were from *Rousettus aegyptiacus.* Sequencing of the VP35, L, and/or NP gene (19, 27, 32, 33) or whole genome sequencing (14, 24) revealed that most isolated bat MARV strains were genetically related to the Angola-like lineage (23%), 1975 Ozolin strain (15%), Gabon strain (12%), Kenya Musoke and Ravn strains (10%), DRC human MARV strain (e.g., 02DRC99) (8%), Uganda MARV (8%), Menglà virus from Rousettus sp. (5%), Chinese Bat9447 (5%), 2012 Ibanda MARV outbreak (5%), 1967 Europe MARV strain (5%), and novel sequences (4%) (Supplementary file 2). The sequences of these isolates were uploaded to GenBank (Supplementary file 2).

### 3.3 Prevalence in domestic animals, NHP and rodents

**Figure 2:**
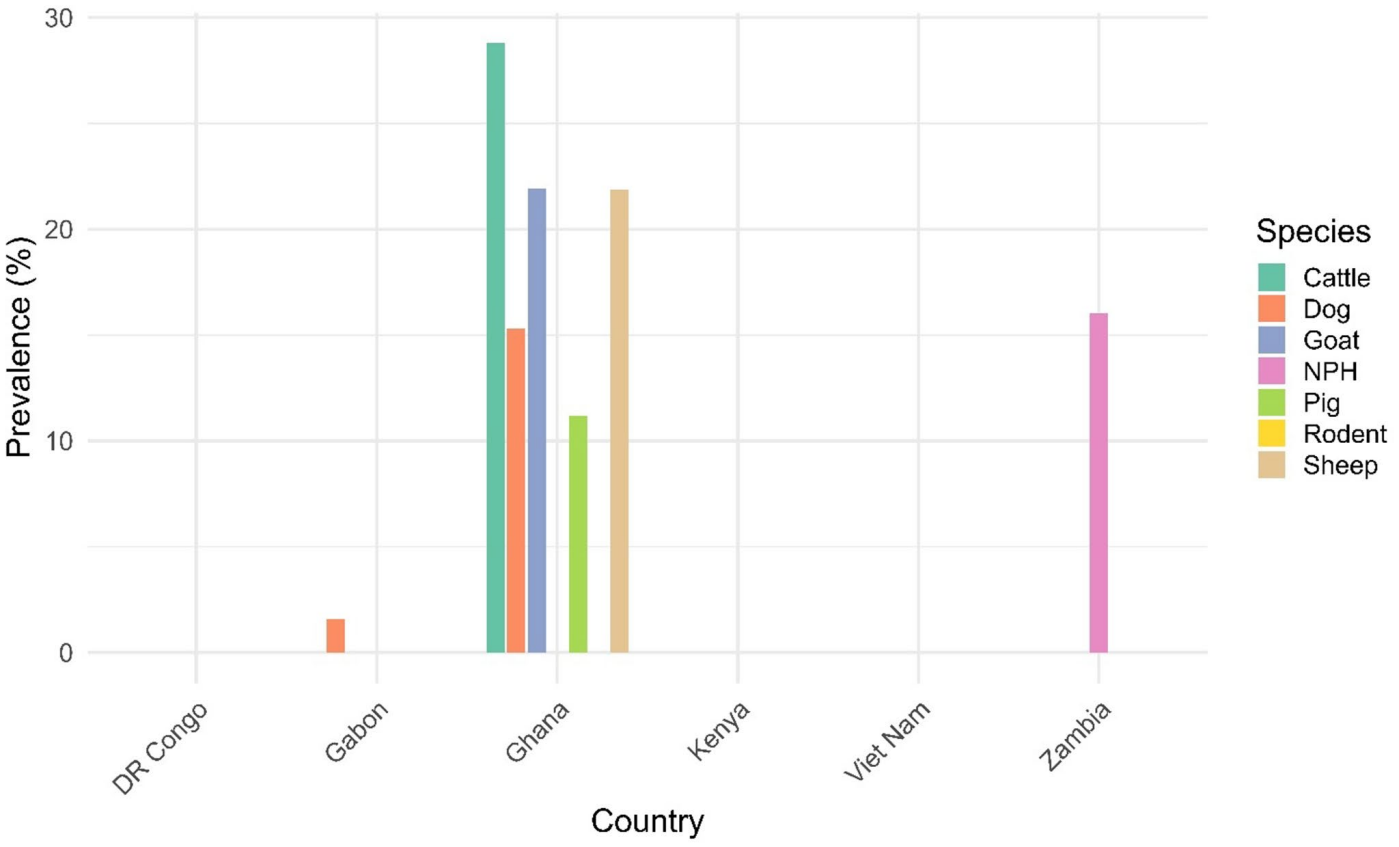
Seroprevalence of Marburg virus antibodies and RNA in livestock, dogs, and rodents.

The six papers exploring MARV antibodies and viral particles were from Gabon (dogs and ruminants), Ghana (livestock and dogs), Kenya and DR Congo (rodents), Zambia (NHP), and Vietnam (pigs). MARV antibodies were identified in dogs and livestock in only two papers from Ghana (34) and Gabon (35), and NHP (36). The identified SP were relatively higher in livestock and dogs in Ghana, where ELISA was used, as compared to Gabon, where the Luminex technique was used (Figure 2). Additionally, Filovirus GP-based ELISA was used for the diagnosis of MARV in NPH. Neither the functionality nor the duration of MARV antibodies in these species was explored in any of the studies. However, all studies hypothesized non-lethal exposure in these species and recommended continuous surveillance to better understand their role in the maintenance of MARV and to inform future pandemic preparedness. MARV viral genes were not amplified in dogs, sheep, goats, and cattle (35), rodents (23, 37), NHP (36), and pigs (38).

Bar plot generated in RStudio (Version 2024.12.0 Build 467) showing MARV antibody and RNA detection in livestock, dogs, and rodents across Gabon, Ghana, Kenya, DR Congo and Vietnam. Antibodies were identified only in dogs and livestock from Ghana and Gabon, with higher seroprevalence in Ghana (in-house I-ELISA) compared to Gabon (Luminex).

## 4 Discussion

This systematic review evaluated the prevalence of MARV in bats, domestic animals, and rodents, the duration of antibodies, and potential routes of viral shedding. This is particular important because African countries continue to face numerous public health challenges, including zoonotic diseases of pandemic potential (39). Yet, research output from the continent remains among the lowest globally (40). Although collaborative research efforts are gradually increasing, they remain insufficient, particularly given the urgent need for funding and effective prevention and control strategies (39, 41). Despite the high case fatality rate of MVD and the fact that most outbreaks occur in Africa (42), much of the research on the virus originates from developed countries (43). These nations, motivated partly by the fear of cross-border transmission due to increased global travel and trade, tend to provide the bulk of available research funding (43).

The COVID-19 pandemic served as a stark reminder that infectious diseases are a global threat, and often emerging from regions with limited surveillance and research capacity. In this context, systematic reviews are especially valuable for African researchers. They synthesize available evidence, identify knowledge gaps, and can inspire new lines of inquiry that attract donor interest. This systematic review contributes to bridging part of the existing research gap, providing a foundation for future investigations and interventions aimed at controlling zoonotic diseases such as MARV infection, which disproportionately affect African populations (42).

This review has identified 11 African countries where outbreaks of Marburg disease in humans have occurred (10, 44, 45). Following the outbreaks, surveys have been conducted in bats, rodents, dogs, and livestock in South Africa, Uganda, Kenya, Zimbabwe, Sierra Leone, the Democratic Republic of the Congo, and Ghana to detect the presence of MARV antibodies and viral particles. These surveillance activities were conducted in high-risk areas, including caves with significant bat and rodent populations, livestock in outbreak zones, and areas where bats and livestock intersect. There is a pressing need for comprehensive surveillance across all affected countries to elucidate the epidemiology of the disease and origins of the outbreaks.

The elevated levels of MARV antibodies and the increased presence of MARV in *Rousettus aegyptiacus* support the hypothesis that this bat species is a primary reservoir of MARV. However, the isolation of MARV and the presence of MARV antibodies in other apparently healthy bat species, including the relatively common *Eidolon helvum*, necessitate consideration of their role in maintaining MARV. The wide range of reported seroprevalence (0.93–53.04%) is biologically plausible and expected given variances in bat ecology, signalment and demography and methodology across the identified studies. Additionally, temporal variation across studies spanning several decades is expected to contribute to fluctuations in seroprevalence due to outbreak cycles and undulating immunity in sampled bats even in the same location.

The duration of MARV antibodies in *R. aegyptiacus* is estimated to be at least 4 months, with potential loss within 3 months of natural horizontal infection or 5 months following vertical transmission (21). This may explain the lower success rates in identifying MARV-specific antibodies in bats. MARV has been isolated from or demonstrated in *R. aegyptiacus* tissues including the kidney and liver (14, 24, 29, 31). However, there is limited evidence conclusively supporting the shedding of the virus through the oral route as the virus has been detected in oral fluid (26). However, the absence of MARV in saliva and its presence in other tissues, as reported by Towner *et al*. (24), led to the hypothesis that transmission through partially eaten fruits, as observed for Ebola and Nipah virus, is unlikely for MARV. Recent experiments show the persistence of the virus for up to 6 h on partially eaten fruits by infected *R. aegyptiacus* (46), which necessitates the need for further investigation into the routes of transmission. Given the elusive duration of MARV antibodies, the brief viremic stage of the virus in bats, and the demonstration of the virus in non-*R. aegyptiacus* bat species, further revealed that other bat species may play a significant role in the maintenance and circulation of MARV.

The hypothesis that livestock, rodents, and dogs contribute to the maintenance of MARV in at-risk areas has recently been explored by four research groups. Although none of these groups detected the virus in these animals, two study groups (34, 35) identified the presence of MARV antibodies in selected livestock and dogs. This hypothesis arises from the unique interactions between dogs, livestock, and at-risk humans in outbreak regions, such as Gabon (35), and wildlife, as observed in Ghana (34). The observed lower MARV antibody detections in livestock and dogs in Gabon, compared to higher levels reported in similar species in Ghana, warrants further investigation. Such variations may reflect differences in ecological conditions, viral circulation, host exposure (47, 48), and sampling strategies and methodologies (34, 35, 49) between the two regions. The detection of MARV antibodies in domestic animals, particularly those in close contact with humans, raises important public health considerations. While the presence of antibodies indicates previous exposure rather than active infection, it nonetheless suggests that these animals may serve as sentinels of viral spillover events or potential intermediate hosts in areas where fruit bats, the natural reservoirs of MARV, are abundant.

None of the included studies performed neutralization assays such as the plaque reduction neutralization test (PRNT), which remains the most specific method for confirming virus-specific antibody responses. However, such assays are rarely applied in MARV research because they require biosafety level-4 containment. Consequently, the observed seropositivity in some studies (27, 34, 35) could, in part, reflect cross-reactivity or low-affinity binding rather than true infection due to shared antigenic regions among filoviruses (50). Furthermore, differences in antigen targets, assay platforms, signal detection thresholds, and cut-off definitions across these serologic tests can lead to variability in sensitivity and specificity between studies. The use of Western Blot (20) and paired sample testing are supplementary approaches that improve confidence in the specificity of the serological tests for MARV antibodies. Experimental work has shown that sera from fruit bats experimentally infected with one filovirus can cross-react with antigens from other members of the Filoviridae family (51). Similar challenges have been reported in other serosurveys of filoviruses (26, 52). Therefore, while antibody detection in wildlife offers valuable clues about potential viral exposure, the absence of confirmatory neutralization testing limits the specificity of these findings and calls for follow-up studies employing assays with higher confirmatory value.

The possibility of domestic animals acting as mechanical or biological bridges for zoonotic transmission cannot outrightly be ruled out, especially in rural communities where livestock, dogs, and humans share habitats and resources. This highlights the importance of adopting a One Health approach to surveillance, integrating animal, human, and environmental health data to understand MARV dynamics. Future research should aim to clarify the epidemiological significance of MARV antibodies in domestic species, whether they indicate incidental exposure or potential roles in viral maintenance; especially when MARV was not demonstrated in any of the exposed species. Strengthening serological surveillance and diagnostic capacity in Africa will be critical to early detection and prevention of future outbreaks. The observed lower MARV antibody detections in livestock and dogs in Gabon detected using Luminex (specificity of 97 across species and target proteins), compared to the higher levels in similar species in Ghana detected using an in-house ELISA (no specificity estimate provided), may primarily be due to differences in the specificity of the tests conducted. However, while the MARV antibodies detected in Gabon may reflect potential exposure to infected individuals, those identified in Ghana are more likely due to repeated exposure to potentially infected bats, as the sampled livestock and hunting dogs frequently interacted with bats in the wild or in forests bordering human settlements. The presence of antibodies in these animals necessitates further investigation into their precise role in the maintenance and transmission of MARV in at-risk and endemic areas.

## 5 Limitations

Only four studies successfully isolated MARV in *Rousettus aegyptiacus* while none used any confirmatory assays to verify MARV antibody specificity, thereby reducing the confidence in the reported serological findings. Both limitations are likely driven by restricted access to BSL-4 laboratory facilities. In addition, substantial heterogeneity in study design, sampled populations, diagnostic approaches, and outcome definitions limits comparability between studies.

## 6 Conclusion

This review highlights the widespread detection of MARV across multiple African countries and species. Evidence supports *Rousettus aegyptiacus* as a primary reservoir, but other bat species may also play critical roles. The recent identification of MARV antibodies in some livestock and dogs warrants further investigation into the role of these animals in the maintenance of MARV. Therefore, there is the need for expanded and targeted multispecies surveillance of MARV in high-risk countries to clarify reservoirs and host distribution as well as the transmission dynamics of MARV.

## Data Availability

The original contributions presented in the study are included in the article/Supplementary material, further inquiries can be directed to the corresponding author.
