# Supplementary material for: Systematic review on Marburg virus prevalence and persistence in animals
Source: Front Vet Sci. 2026 Feb 4;13:1756506. doi: 10.3389/fvets.2026.1756506 (PMC12913193; doi:10.3389/fvets.2026.1756506)
Supplement: Supplementary file 1 [file Table_1.DOCX]

| **Section and Topic** | **Item #** | **Checklist item** | **Location where item is reported** |
| --- | --- | --- | --- |
| **TITLE** | | |  |
| Title | 1 | Systematic Review on Marburg Virus Prevalence and Persistence in Animals | Title page |
| **ABSTRACT** | | |  |
| Abstract | 2 | Marburg Virus Disease (MVD) is a fatal zoonotic disease of humans and non-human primates caused by the Marburg virus (MARV) of the *Filoviridae* family, and presenting as haemorrhagic fever with a high fatality rate. Egyptian fruit bats, *Rousettus aegyptiacus*, are the principal natural reservoir, with evidence linking them to most human outbreaks. This systematic review evaluated the prevalence of MARV in bats, domestic animals, and rodents, as well as the duration of antibodies and potential routes of viral shedding. A comprehensive search of six (6) scientific databases identified thirty (30) studies meeting the inclusion criteria. In bats, seroprevalence ranged from less than 1% to about 54% while MARV genes were detected in 0.8% – 3% of samples. MARV antibodies persisted for up to eleven (11) months in naturally infected bats, while induced or maternal antibodies declined within five (5) months.  Apart from *Rousettus aegyptiacus*, occasional seropositivity was detected in other bat species such as *Epomops franqueti*, *Micropteropus pusillus*, *Hypsignathus monstrosus*, and *Eidolon helvum*, whereas MARV particles were observed in *Rousettus aegyptiacus* and *Hipposideros* spp. Even though viral genes were undetected in domestic animals, non-human primates (NPH) and rodents, antibodies were reported in dogs and livestock, and NPH in Ghana and Gabon and Zambia respectively indicating a higher probability of non-lethal MARV exposure in these species.  These findings confirm *Rousettus aegyptiacus* as the primary reservoir but suggest that other bats and domestic animals may contribute to the natural maintenance of MARV. Expanded multispecies surveillance in high-risk regions is essential to clarify reservoirs, host distribution, and transmission dynamics. Understanding these patterns is critical for designing targeted interventions to reduce spillover risk to humans. | Abstract section |
| **INTRODUCTION** | | |  |
| Rationale | 3 | Egyptian fruit bats (*Rousettus aegyptiacus*) are recognized as the primary natural reservoir of MARV, and human outbreaks are largely driven by spillover from bats followed by human-to-human transmission. A synthesis of evidence on MARV prevalence across animal hosts, antibody persistence, and shedding routes is essential to better understand MARV ecology and transmission dynamics. | Introduction paragraph 2 |
| Objectives | 4 | 1. To review the prevalence of MARV in bats, rodents, non-human primates, and domestic animals from the first recorded outbreak to May 2025. 2. To review the duration of MARV antibodies and routes of viral shedding in identified animal species. | Introduction paragraph 2 |
| **METHODS** | | |  |
| Eligibility criteria | 5 | Studies were included if they   1. were written in English; 2. investigated MARV prevalence in bats, rodents, non-human primates, or livestock; 3. reported on MARV antibody duration; 4. examined virus isolation or shedding routes in animals; and 5. were published before May 2025. | Methods section 2.1 |
| Information sources | 6 | Databases searched included PubMed, Google Scholar, Web of Science, African Journals Online (AJOL), Scopus, ScienceDirect, and grey literature sources. Searches were initially conducted between March and April 2025 and again in January 2026 (during the review process). | Methods section 2.1 |
| Search strategy | 7 | A database-specific search strategy was developed using combinations of keywords and MeSH terms related to “Marburg virus,” “bat,” “livestock,” “domestic animals,” “non-human primates,” and “prevalence.” Searches were restricted to English-language articles and excluded preprints, reviews, and meta-analyses. Searches were conducted on March 18 and repeated on April 26, 2025. All records were managed using Mendeley Reference Manager (version 2.130.2). | Methods section 2.1, paragraph 1 |
| Selection process | 8 | Duplicates were identified using Mendeley and manual verification. Titles and abstracts were screened against predefined inclusion criteria. Full-text screening was conducted for eligible studies. | Methods section 2.1, paragraph 2 |
| Data collection process | 9 | Data were extracted using a structured questionnaire developed in KoboToolbox. Extracted information included study metadata, species sampled, diagnostic methods, outcomes, and risk of bias. | Methods section 2.2 |
| Data items | 10a | Extracted data included   1. study metadata (authors, year, country, funding); 2. species and sample types; 3. diagnostic approaches and outcomes; 4. viral isolation and shedding information; and 5. risk of bias assessment using JBI tools. | Methods section 2.2 |
|  | 10b | Additional contextual information, including the effects of age and sex on MARV prevalence, was collected where available. Ambiguous information was clarified by consensus among authors. | Methods section 2.2 |
| Study risk of bias assessment | 11 | Risk of bias was assessed using the Joanna Briggs Institute (JBI) critical appraisal tools. Each study was independently assessed by two reviewers. | Methods section 2.2 |
| Effect measures | 12 | Prevalence was categorized as molecular prevalence (PCR- or sequencing-based detection) or serological prevalence (ELISA, immunofluorescence, Luminex, or bioplex assays, etc). | Methods section 2.1 |
| Synthesis methods | 13 | Data were analysed using RStudio (version 2024.12.0). Mixed-effects meta-regression models were fitted using restricted maximum likelihood estimation. Heterogeneity was quantified using τ², I², and Cochran’s Q statistics, and the proportion explained by moderators was assessed using R². Descriptive summaries were presented as tables and figures. | Methods section 2.2; Supplementary File 2 |
| Reporting bias assessment | 14 | Disagreements during screening or data extraction were resolved by a third reviewer. | Methods section 2.1 |
| Certainty assessment | 15 | Certainty of evidence was not formally graded (e.g., using GRADE) due to substantial heterogeneity in study designs, diagnostic methods, and outcome measures. Instead, study credibility was appraised using the Joanna Briggs Institute (JBI) critical appraisal tools. |  |
| **RESULTS** | | |  |
| Study selection | 16a | A total of 16,735 records were identified. After screening and duplicate removal, 30 full-text articles were assessed, and 26 studies reporting MARV prevalence were included. Five studies additionally reported antibody duration or viral isolation. | Results paragraph 1 |
|  | 16b | Studies were excluded due to duplication, preliminary reporting, or lack of relevance to inclusion criteria. For instance,   1. Report of studies that have been already published: e.g., Marburg virus could come from fruit bats, [Godefroy Macaire Chabi and Christina Scott](https://www.proquest.com/docview/2707693112?pq-origsite=gscholar&fromopenview=true&sourcetype=Newspapers), [**SciDev.net - Health**](https://www.proquest.com/docview/2707693112?pq-origsite=gscholar&fromopenview=true&sourcetype=Newspapers)**; Oxford**. 05 Sep 2007. [Marburg virus could come from fruit bats - ProQuest](https://www.proquest.com/docview/2707693112?pq-origsite=gscholar&fromopenview=true&sourcetype=Newspapers). It writes about doi 10.1371/journal.pone.0000764 2. Preliminary report of studies that were later published: e.g., Deadly Marburg Virus Found in Sierra Leone Bats \| CDC Online Newsroom \| CDC. It discusses preliminary results from 10.1038/s41467-020-14327-8 3. Unrelated to inclusion criteria: e.g., 10.3389/fviro.2021.759655.this paper discusses innate immunity in ERB which is an inclusion criterion for our review |  |
| Study characteristics | 17 | Characteristics of all included studies are provided in Supplementary File 2. | Methods section 2.2; Supplementary File 2 |
| Risk of bias in studies | 18 | Risk of bias assessments for each study are reported in Supplementary File 2. | Result section 3.1, 3.2, and 3.3; Supplementary File 2 |
| Results of individual studies | 19 | Extracted data and individual study outcomes are reported in Supplementary File 2. | Result section 3.1, 3.2, and 3.3; Supplementary File 2 |
| Results of syntheses | 20a | Results of data synthesis are presented as tables and figures in the main text and Supplementary File 2. | Result section 3.1, 3.2, and 3.3; Supplementary File 2 |
|  | 20b |  |  |
|  | 20c |  |  |
|  | 20d |  |  |
| Reporting biases | 21 | Potential reporting biases were assessed using funnel plot asymmetry and regression-based methods. |  |
| Certainty of evidence | 22 | A formal GRADE assessment was not performed due to substantial heterogeneity across study designs, outcomes, and diagnostic methods. |  |
| **DISCUSSION** | | |  |
| Discussion | 23a | Results are interpreted in the context of existing evidence on MARV ecology and transmission. |  |
|  | 23b | Limitations include the absence of confirmatory neutralization assays for antibody specificity and the limited success of virus isolation attempts. | Limitation |
|  | 23c | Substantial heterogeneity in study design, populations, diagnostic approaches, and outcome definitions limits comparability across studies. |  |
|  | 23d | The findings highlight *R. aegyptiacus* as the primary MARV reservoir while suggesting potential roles for other bats and domestic animals, underscoring the need for expanded multispecies surveillance. |  |
| **OTHER INFORMATION** | | |  |
| Registration and protocol | 24a | This review was not registered in PROSPERO because prevalence-based animal studies were not eligible at the time of protocol development. |  |
|  | 24b |  |  |
|  | 24c |  |  |
| Support | 25 | No specific funding was received for this study. |  |
| Competing interests | 26 | The authors declare no competing interests |  |
| Availability of data, code and other materials | 27 | 1. Supplementary File 1 contains the PRISMA checklist. 2. Supplementary File 2 includes extracted data, datasets used for all analyses, and analytic code. 3. All materials are publicly available online at: |  |

*From:*  Page MJ, McKenzie JE, Bossuyt PM, Boutron I, Hoffmann TC, Mulrow CD, et al. The PRISMA 2020 statement: an updated guideline for reporting systematic reviews. BMJ 2021;372:n71. doi: 10.1136/bmj.n71. This work is licensed under CC BY 4.0. To view a copy of this license, visit <https://creativecommons.org/licenses/by/4.0/>
